# Transmission of toxigenic *Corynebacterium diphtheriae* by a fully immunised resident returning from a visit to West Africa, United Kingdom, 2017

**DOI:** 10.2807/1560-7917.ES.2018.23.39.1700681

**Published:** 2018-09-27

**Authors:** David Edwards, Dianne Kent, Caroline Lester, Colin Stewart Brown, Michael E. Murphy, Nicholas M. Brown, Olajumoke Sule, Alexandra Itani, Meera Chand, Amy Trindall, Callum Pearson, Iain Roddick, Norman K. Fry, Jorg Hoffmann, Nalini Iyanger, Laurence Kemp, Joanne White, Babak Javid, Isobel D. Ramsay, Dominik Zenner, Aliko Ahmed, Gayatri Amirthalingam, Sultan Salimee, David Litt, Mark Reacher

**Affiliations:** 1 East of England Health Protection Team, Public Health England, Thetford, United Kingdom; 2 National Infection Service, Public Health England, London, United Kingdom; 3 Department of Microbiology, Cambridge University Hospitals NHS Foundation Trust, Cambridge, United Kingdom; 4 PHE Public Health Laboratory Cambridge, Public Health England, Cambridge, United Kingdom.; 5 Granta Medical Practices, Cambridge, United Kingdom; 6 NIHR Health Protection Research Unit in Respiratory Infections, Public Health England, London, United Kingdom; 7 Field Epidemiology Service, Public Health England, Cambridge, United Kingdom; 8 Department of Medicine, University of Cambridge School of Clinical Medicine, University of Cambridge Hospitals Trust, Cambridge, United Kingdom; 9 Immunisation, Hepatitis and Blood Safety Department, National Infection Service, Public Health England, London, United Kingdom

**Keywords:** Corynebacterium diphtheriae, toxigenic, outbreak, corynebacterium infection, vaccine-preventable diseases, health protection

## Abstract

In early 2017, a United Kingdom (UK)-born person in their 20s presented with a skin ulcer on the foot 3 weeks after returning from Ghana. The patient had last received a diphtheria-containing vaccine in 2013, completing the recommended course. MALDI-TOF of a cutaneous swab identified *Corynebacterium diphtheriae*. Real-time PCR ascertained the species and presence of the diphtheria toxin gene. An Elek test confirmed toxigenicity. The isolate was macrolide sensitive and penicillin resistant. The local Public Health England (PHE) Health Protection Team obtained the patient’s clinical history and traced contacts to inform appropriate public health action. One close contact (in their early 80s with uncertain immunisation status who had not recently travelled) had a positive throat swab for toxigenic *C. diphtheriae* and reported a history of mild coryzal symptoms. Multilocus sequence typing revealed that strains from the index case and contact had Sequence Type 463. Diphtheria is extremely rare in the UK due to high vaccine coverage and this is the first documented transmission in 30 years. Clinicians and laboratory staff should remain highly suspicious of lesions in overseas travellers, even when patients are fully vaccinated. Older individuals who might not have completed a full immunisation course may have higher diphtheria susceptibility.

## Introduction

Diphtheria is a disease caused by toxin-producing strains of *Corynebacterium diphtheriae* and, in some cases, also by *C. ulcerans* bacteria [1]. It is transmitted from person to person through close physical and respiratory contact. The early signs of respiratory diphtheria are sore throat, loss of appetite, and slight fever with classic symptoms including the development of a white (turning to grey) membrane in the throat and on the tonsils [2]. Severe cases of diphtheria may develop a swollen neck and obstructed airway. Cutaneous diphtheria lesions can be highly variable, including presentation as painful ulcers covered with a dark pseudomembrane, serous oozing lesions, or dry, nearly healed, scaly lesions [3].

Infection with toxigenic diphtheria is rare in the United Kingdom (UK); in 2016 there were six cases infected with toxigenic strains of corynebacteria reported in England, four *C. diphtheriae* and two *C. ulcerans* [4]. Diphtheria vaccination is highly effective and immunisation coverage of the UK population remains high [5,6]. A review of UK diphtheria cases between 1986 and 2008 found the majority had mild infections with the affected individuals either only being partially immunised, or fully immunised adults with waning immunity [7]. Sero-epidemiological studies have demonstrated waning immunity among adults in the UK and other Western European countries, although this is not likely to present a risk of widespread disease re-emergence [2].

In February 2017, the Public Health England (PHE) East of England Health Protection Team (HPT), which conducts local disease surveillance and outbreak investigations, was advised that a potentially toxigenic isolate of *C. diphtheriae* had been isolated from a cutaneous lesion of an East of England resident. This article summarises the public health investigations and actions taken, and discusses their implications for future diphtheria outbreak investigations and policy.

## Methods

### Laboratory analyses

Skin swabs were inoculated on blood and cystine-lactose-electrolyte deficient (CLED) agar plates and incubated for 48 hours at 37 °C in 5% carbon dioxide. Throat swabs were inoculated on Staph/Strep selective agar and incubated for 24 hours at 37 °C in room air. Samples from patients with a request for *C. diphtheriae* were additionally inoculated on Hoyle’s tellurite medium and incubated for 48 hours at 37 °C in room air and evaluated for growth of indicative grey/black organisms. Positive growth in culture was identified using Matrix Assisted Laser Desorption/Ionization–Time of Flight mass spectrometry (MALDI-TOF; Bruker Corporation, Massachusetts, United States). Organisms identified as *C. diphtheriae* by MALDI-TOF were confirmed as *C. diphtheriae* containing the diphtheria toxin gene by real-time PCR directed against the *rpoB* gene and fragment A of the diphtheria toxin gene [8]. They were confirmed as toxigenic using the modified Elek test [9]. Sensitivity testing was performed using E-tests (bioMérieux, Basingstoke, UK) inoculated on ISO-Sensitest agar supplemented with 5% defibrinated horse blood as per British Society for Antimicrobial Chemotherapy (BSAC) guidelines [10]. Resistance was tested against the following antibiotics: ciprofloxacin, clindamycin, doxycycline, erythromycin, linezolid, penicillin, rifampicin and vancomycin. The cut-off values used to evaluate resistance were the European Committee on Antimicrobial Susceptibility Testing (EUCAST) Clinical Breakpoints for *Corynebacterium* spp. [11]. Organisms were further characterised by multilocus sequence typing (MLST; with reference to www.pubmlst.org/cdiphtheriae/) and biovar determination (API Coryne test, bioMérieux, Basingstoke, UK) [12]. All agars described were obtained from Oxoid, Oxford, UK.

### Management and investigation of cases and contacts 

The management of suspected or confirmed diphtheria cases and their close contacts was conducted according to the PHE’s national guidelines [13]. In general, contacts were advised to seek medical attention if they become unwell and to self-monitor for 10 days from date of exposure. They were offered chemoprophylaxis and diphtheria toxoid containing vaccine in line with national guidance via their own GP services [13]. Demographical information (age and sex) and exposure details were collected routinely as part of the identification/interview process for close contacts. Close contacts were defined as those sleeping in the same household as the index case, kissing/sexual contacts, healthcare workers who had direct exposure to respiratory droplets or secretions, or dressed cutaneous lesions, without appropriate personal protective equipment. Further information on the vaccination history of cases and close contacts was obtained via a questionnaire. The travel history of a second case was obtained by direct interview by the HPT and entered onto clinical notes.

## Results

### Detection, care and investigation of the index case

The index case (Case 1) was a 20-year-old office worker who returned on 10 January 2017 from a three-week trip volunteering at an orphanage in Ghana. The patient had received all five doses of a diphtheria-containing vaccine according to the UK schedule with three doses in the first year of age, and booster dose at age 3 and 14 years. After returning, she attended her GP surgery on several occasions (11, 16 and 18 January 2017) with suspected urinary tract infection (UTI) which settled with antibiotic therapy (flucloxacillin and nitrofurantoin) plus gastrointestinal symptoms and skin lesions diagnosed as infected bite wounds. On 30 January 2017 the case presented to her GP surgery with tonsillitis (onset 27 January 2017) and two non-healing ulcers on her right foot. Superficial swabs of the skin lesions were taken by the GP for microbial culture, but because the patient was systemically well she was not prescribed any antibiotics on this occasion.

Skin swabs taken from the Case 1 on 30 January 2017 grew a mixture of bacterial colonies. MALDI-TOF identified the most abundant organism as *C. diphtheriae* on 09 February 2017. The hospital microbiology laboratory reported isolation of *C. diphtheriae* to the local PHE HPT and the PHE national clinical leads for diphtheria, and the isolate was referred to the PHE national diphtheria reference laboratory for further analysis.

Case 1 was interviewed by the HPT on 09 February 2017 to undertake a risk assessment of the likelihood of toxigenic disease and identify close contacts. She was advised to self-isolate until the results from toxigenicity testing on the isolate were available as she was considered clinically stable and did not meet criteria for anti-toxin administration. On the same day a throat swab was taken from case 1 at her GP practice who prescribed erythromycin treatment. 

The following day (10 February 2017), using real-time PCR, the national reference laboratory confirmed that the isolate was *C. diphtheriae* and that it possessed the diphtheria toxin gene. 

On 11 February 2017, Case 1 was given a diphtheria toxoid-containing booster vaccination. The case was advised to continue self-isolation until two sets of negative clearance swabs (taken from nose, throat and skin lesions (if not healed)) were obtained 24 hours and 72 hours after completion of antibiotics, i.e. 24 February 2017 and 27 February 2017. As for the throat swab taken on 09 February 2017, throat and nose swabs taken from Case 1 on 24 and 27 February 2017 were all culture negative for *C. diphtheriae* (Figure). The cutaneous lesions had already healed by the end of the antibiotic treatment, so no further cutaneous swabs were obtained.

**Figure:**
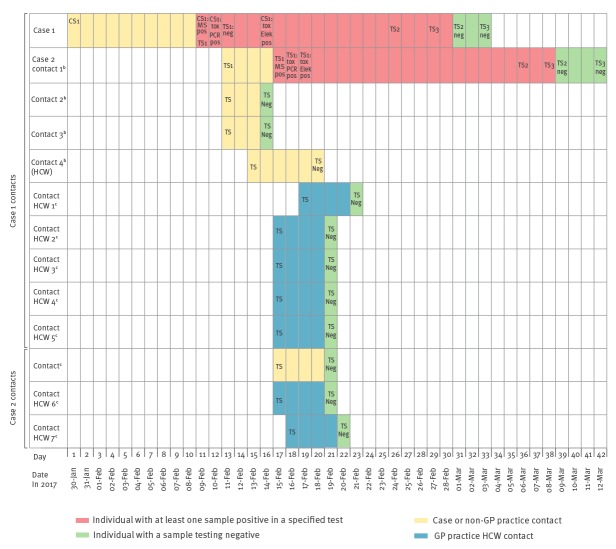
Timeline of swab samples and test results for toxin-positive *Corynebacterium diphtheriae* cases and close contacts^a^, East of England, January–March 2017 (n = 13)

CS: cutaneous swab; GP: general practitioner; HCW: healthcare worker; MS pos: sample positive for *C. diphtheriae* by Matrix Assisted Laser Desorption/Ionization–Time of Flight (MALDI-TOF) mass spectrometry; neg: negative for *C. diphtheriae*; tox Elek pos: isolate of *C. diphtheriae* confirmed to be toxigenic by modified Elek test; tox PCR Pos: isolate of *C. diphtheriae* confirmed to possess the toxin gene by PCR; TS: throat swab (numbered in event of more than one swab taken).

^a^ Close contacts were defined as those sleeping in the same household as the index case, kissing/sexual contacts, healthcare workers who had direct exposure to respiratory droplets or secretions, or dressed cutaneous lesions, without appropriate personal protective equipment.

^b^ Close contact who was not a GP practice HCW contact.

^c^ Close contact who was a GP practice HCW contact.

The *C. diphtheriae* isolate from Case 1 was confirmed to be expressing diphtheria toxin (i.e. toxigenic) on 14 February 2017. The results of further testing of the isolate in late February showed it to be resistant to penicillin (MIC 1.0 mg/L) but susceptible to erythromycin.

### Tracing and follow-up of contacts

Following confirmation on 10 February 2017 that the isolate from Case 1 was positive for a *C. diphtheriae* strain harbouring a toxin gene, active contact tracing was undertaken by the HPT according to PHE national guidelines [12]. Further details on the clinical history of the case were obtained from the GP practice clinical record as part of the investigation. On 10 February 2017, three close contacts (family and household) were identified. On 11 February 2017, as per national guidelines the close contacts were swabbed (nasopharyngeal, throat, and skin if any lesions present; Figure). They were also prescribed erythromycin chemoprophylaxis and arrangements were made for booster diphtheria immunisation at the earliest opportunity. 

One of the close contacts was an 81-year-old UK-born female with uncertain immunisation status who had not recently travelled. She reported a history of mild coryzal symptoms and her throat swab was subsequently shown to be positive for *C. diphtheriae* by culture and MALDI-TOF. Further testing (by PCR and modified Elek test) confirmed that this isolate was also toxigenic (Figure). This person was designated Case 2. She was initially prescribed erythromycin as chemoprophylaxis on 12 February 2018 along with the other close contacts, but did not tolerate it well due to gastrointestinal symptoms and was empirically switched to benzylpenicillin chemoprophylaxis by intramuscular injection on 14 February 2017. This was 6 days prior to the penicillin resistance result communication to the Incident Control Team (ICT) on 20 February 2017. Case 2 initially did not wish to take further antibiotics and eventually started her prescribed treatment with clarithromycin on 21 February 2017. She was advised to self-isolate until completion of treatment and the finding of two consecutive negative clearance throat swab samples (taken on 6 and 8 March 2017). An additional close contact (family), a paediatric nurse, was identified on 12 February 2017 and excluded from clinical duties. This person attended their GP on 13 February 2017 to be swabbed, prescribed chemoprophylaxis and receive a booster diphtheria immunisation.

On 15 February 2017 a further eight close contacts were identified, five of Case 1 and three of Case 2 (Table); these included seven GP practice healthcare workers (Figure). All received appropriate screening, chemoprophylaxis and booster diphtheria immunisation. The seven healthcare workers were excluded from clinical work until negative throat swabs had been obtained. This had a substantial impact on the GP practice, which had to cancel a number of patient appointments due to unexpected staff shortage. All throat swabs screened negative for *C. diphtheriae*. The immunisation status of cases (two women) and close contacts (seven women and four men) was investigated as part of the response to consider the value of booster vaccination as part of the post exposure prophylaxis. This identified that only one of the two cases had received five doses of diphtheria toxoid containing vaccine, and only four of 11 close contacts, although vaccination history proved unobtainable for four close contacts, all of whom were healthcare workers.

**TABLE:** Demographics and immunisation status with diphtheria toxin vaccine for toxin-positive *Corynebacterium diphtheriae* cases (n = 2) and their close contacts^a^ (n = 11), East of England, January–March 2017

Relationship to case		Age group in years	PPE worn	Reported doses of diphtheria containing vaccine received^b^
Case 1 contacts	Case 1	11–20	NA	Five
	Case 2 Non-GP practice contact 1	81–90	NA	No record of any doses received
	Non-GP practice contact 2	21–30	No	Five
	Non-GP practice contact 3	51–60	No	No record of any doses received
	Non-GP practice contact 4; HCW	41–50	No	Five
	GP practice contact HCW 1	31–40	No	No vaccination information obtained
	GP practice contact HCW 2	31–40	Unknown	No vaccination information obtained
	GP practice contact HCW 3	41–50	No	No vaccination information obtained
	GP practice contact HCW 4	51–60	No	Five
	GP practice contact HCW 5	51–60	No	Five
Case 2 contacts	Non-GP practice contact	41–50	No	One
	GP practice contact HCW 6	51–60	No	No vaccination information obtained
	GP practice contact HCW 7	41–50	No	One

HCW: healthcare worker; NA: not applicable; PPE: personal protective equipment.

^a^ Close contacts were defined as those sleeping in the same household as the index case, kissing/sexual contacts, healthcare workers who had direct exposure to respiratory droplets or secretions, or dressed cutaneous lesions, without appropriate personal protective equipment.

^b^ Recorded doses have been obtained from reviewing medical records or self-reported via a questionnaire.

### Typing results

The bacterial isolates from Case 1 and Case 2 were both subsequently shown to be biovar mitis strains. MLST showed that they possessed the same novel sequence type (designated ST493; allelic profile 28,2,54,30,3,3,3), supporting a hypothesis of a single source and the epidemiological evidence of transmission.

## Discussion

This is the first documented case of transmission of diphtheria within the UK in 30 years. The rarity of diphtheria in the country is attributed to an effective immunisation programme with sustained high vaccine coverage in the population. The last recorded transmission of toxigenic *C. diphtheriae* occurred in 1986 in a family of recent immigrants from Bangladesh [7]. In that instance, the cases were unimmunised, and both cutaneous and respiratory presentations were identified [7]. In the current event, the index case had a cutaneous infection. It is notable that transmission occurred despite the fact that this person was vaccinated.

In terms of risk factors for diphtheria in the UK, there have been documented cases following zoonotic exposure to *C. ulcerans* from companion animals [4,14]. *C. ulcerans* infection is also a known occupational hazard following exposure to agricultural livestock [15]. For toxigenic *C. diphtheriae* however, the main risk factors are travel to an endemic country, close contact with a diphtheria case and being unvaccinated or under-vaccinated. 

Indeed, there remain a number of countries, including Ghana, where diphtheria is endemic, despite an improving immunisation coverage with diphtheria containing vaccine (DTP) [16]. In our situation, the index case had travelled to such a country, but had received a primary course of three doses of diphtheria toxoid containing vaccine plus two booster doses of vaccine as per the current vaccination schedule [17]. This incident highlights that cutaneous infection can occur in fully vaccinated individuals and this presents a risk for transmission to people without up-to-date immunisation or with an impaired or waning immune response. 

The presentation of skin infections due to toxigenic *C. diphtheriae* has been widely documented [18]. Between 2007 and 2013 there were five imported cutaneous toxigenic *C. diphtheriae* infections in England [19]. Clinicians and laboratory staff should retain a high index of suspicion of *C. diphtheriae* and *C. ulcerans* in skin lesions from overseas travellers, even when patients are fully vaccinated. This has important implications for use of personal protective equipment (PPE) by clinicians to whom patients may first present. In addition, *C. diphtheriae* and *C. ulcerans* are classed as Hazard Group 2 organisms and laboratory workers should determine their diphtheria antibody status, especially if they will potentially handle toxigenic corynebacteria [17]. It is notable that some clinicians exposed to the cases described in this report had uncertain immunisation status with regard to diphtheria.

The healthcare close contacts in the current event included GP practice staff with one GP practice impacted by exclusion at short notice of five staff from clinical duties leading to cancellation of patient appointments. The close contacts also included a specialist paediatric healthcare worker who could come into contact with unimmunised infants. 

There was no dissemination outside of a close family contact whose household the index case stayed at overnight; this echoes the experience of investigators of a household outbreak of toxigenic diphtheria in Norway in 2008 where there were three cases in unimmunised individuals within the same family [20]. These cases developed a confluent, thick yellow membrane across their tonsils and peritonsillar tissue. In addition the index case developed the classic ‘bulls neck’ appearance. In the present report, no cases of classic diphtheria occurred. 

The current transmission event highlights the importance of early diphtheria case ascertainment and a timely health protection response to protect vulnerable individuals. In this respect, the more widespread availability of MALDI-TOF in routine diagnostic laboratories offers an opportunity for improved and timelier identification of *C*. *diphtheriae* and *C. ulcerans*. This is also a welcome development in terms of the need to ensure diphtheria diagnostic capacity across all European Union/European Economic Area countries in light of important gaps identified by the European Centre for Disease Prevention and Control (ECDC) [21]. Rapid real-time PCR for the detection of potentially toxigenic isolates also allows treatment and public health measures to be initiated more quickly than if relying on phenotypic toxigenicity testing alone.

Case 2 was initially prescribed erythromycin as chemoprophylaxis as a close contact before she was identified as a case – PHE recommended agents for chemoprophylaxis are either erythromycin (7 days) or, if more easily administered, a single intramuscular (IM) dose of benzylpenicillin. The case did not tolerate erythromycin due to gastrointestinal symptoms therefore benzylpenicillin was administered. At the time chemoprophylaxis was offered the resistance to penicillin of the *C. diphtheriae* strain had not been determined. Subsequently when offered treatment for diphtheria the case was reluctant to take further antibiotics following her experience of erythromycin. The national guidelines indicate that alternative macrolide antibiotics are available including clarithromycin and azithromycin.

## Conclusion

This transmission event underlines the importance of timely investigation of cases and contact tracing to avoid further spread of disease. Clinicians and clinical laboratories must maintain a high degree of awareness of diphtheria, including the occurrence of skin lesions, especially in persons with a history of travel to countries where diphtheria is endemic.
