# Authors’ correction for Euro Surveill. 2018;23(39)

**DOI:** 10.2807/1560-7917.ES.2018.23.42.1810181

**Published:** 2018-10-18

**Authors:** Eurosurveillance editorial team

**Affiliations:** 1 European Centre for Disease Prevention and Control (ECDC), Stockholm, Sweden

As requested by the authors of the article ‘Transmission of toxigenic Corynebacterium diphtheriae by a fully immunised resident returning from a visit to West Africa, United Kingdom, 2017’ which was published on 27 September 2018, a change was made to the abstract. In the abstract, the Sequence Type stated as 463 should have been 493. ‘Sequence Type 463’ was corrected to ‘Sequence Type 493’ on 15 October 2018.

